# Nachhaltige StadtGesundheit: konzeptionelle Grundlagen und aktuelle Initiativen

**DOI:** 10.1007/s00103-020-03187-8

**Published:** 2020-07-08

**Authors:** Alf Trojan, Rainer Fehr

**Affiliations:** 1 Institut für Medizinische Soziologie, Universitätsklinikum Hamburg-Eppendorf, Martinistr. 52, 20246 Hamburg, Deutschland; 2 Fakultät für Gesundheitswissenschaften, Universität Bielefeld, Bielefeld, Deutschland

**Keywords:** Nachhaltigkeit, Gesundheitsförderung, Gesundheitspolitik, Gesundheit in allen Politikbereichen, Gesundheitsfördernde Gesamtpolitik, Sustainability, Health promotion, Health policy, Health in all policies, Healthy public policy

## Abstract

Die Wechselwirkungen zwischen Stadt und Gesundheit werden schon seit der Antike thematisiert. Seit Ende der 1970er-Jahre wird die Bedeutung außermedizinischer Determinanten der Gesundheit wieder verstärkt in den Blick genommen. Ziel des Beitrags ist es, die Entstehung wichtiger neuer Konzepte der letzten 40 Jahre nachzuzeichnen, ihr Verhältnis zueinander zu klären und darauf aufbauend das Konzept der „Nachhaltigen StadtGesundheit“ (Sustainable Urban Health) darzustellen. Dazu erfolgten eine Sekundäranalyse und die Interpretation relevanter Dokumente und einschlägiger Literatur.

Nach einer Einführung mit illustrativen Beispielen der Behandlung des Themas „Stadt und Gesundheit“ wird gezeigt, dass die Weltgesundheitsorganisation (WHO) seit den 1970er-Jahren mit den Konzepten „multi- bzw. intersektorale Aktionen“, „gesundheitsfördernde Gesamtpolitik“ und „Gesundheit in allen Politikbereichen“ maßgeblich den neuen Public-Health-Diskurs unterfüttert hat. „Nachhaltige StadtGesundheit“ steht in dieser Tradition und wird hier als Programm der Blickfelderweiterung und des Brückenbaus zwischen Disziplinen und Sektoren charakterisiert. Folgend wird es durch eine Hamburger Initiative exemplarisch illustriert.

Die WHO-Konzepte stellen eher unterschiedliche Akzentuierungen dar, als dass sie sich substanziell unterscheiden. Health in All Policies (HiAP) liegt auf der Linie der Menschrechtsdeklaration und der Nachhaltigkeitsziele der Vereinten Nationen; es hat den Eingang sowohl in die WHO- als auch in die Politik der Europäischen Gemeinschaft geschafft. Für das Ziel von „Nachhaltiger StadtGesundheit“, synchron Gesundheit und Nachhaltigkeit in der Stadtgesellschaft zu fördern, ist weitere Entwicklungsarbeit zu leisten.

## Abstract (en)

The interactions between cities/settlements and health have been an issue of discussion since antiquity. Since the late 1970s, there has been renewed interest in the role of nonmedical determinants of health. This paper aims to retrace the development of relevant new concepts during the last 40 years, clarify their mutual relationships, and from this background present the concept of sustainable urban health. For this purpose, a secondary analysis is conducted and relevant documents and literature are being interpreted.

After an introduction using illustrative examples of urban health, the paper describes how the WHO has provided crucial input to a renewed debate on public health since the 1970s, based on concepts such as “multi-/intersectoral action,” “healthy public policy,” and “health in all policies.” Sustainable urban health, tying in with this tradition, is characterized as a program of expanding the horizon and of building bridges between disciplines and societal sectors. This is exemplified by a local initiative in the city of Hamburg.

Rather than being substantially different, the WHO concepts may be seen as representing various shades of emphasis. Health in all policies (HiAPs) are in line with the UN Declaration of Human Rights and the UN Sustainable Development Goals; the concept succeeded in being integrated into both WHO and EU policy making. Additional work is required for the sustainable urban health goal to synchronously promote health and sustainability in urban societies.

## Der umfassende Blick auf urbane Gesundheit in historischen Beispielen

Policy, Policey und Politik haben ihren etymologischen Ursprung in dem griechischen Begriff Politeia, der die gute Ordnung im Gemeinwesen bzw. den Stadtstaaten bezeichnete. Seit dem 17. Jahrhundert zielten die „policeylichen“ Regelungen auf Wirtschaftlichkeit, Sicherheit und auch auf die Gesundheit der Bevölkerung. Höhepunkt war das sechsbändige Werk über das System einer vollständigen medizinischen Policey (1779–1819) des kaiserlichen Leibarztes und hohen Medizinalbeamten Johann Peter Frank [1]. Darin ging es um die Bevölkerungsgesundheit (Public Health). Aufgabe des Regenten war es danach, die Ratschläge der Medizin auf lokaler Ebene umzusetzen: „Die Medizinalpolicey des 18. Jahrhunderts bemühte sich um eine Verstetigung und Ausweitung der präventiven Schutzvorkehrungen im öffentlichen Raum. Eine verbesserte Entsorgung von Fäkalien, Abwässern und Müll, die Sicherung der Frischwasserzufuhr durch Gemeinschaftsbrunnen, das Reinhalten der Luft, des Wassers und des Bodens durch die Trennung von Gewerbegebieten, Friedhöfen und Wohnsiedlungen, die regelmäßige Durchführung von Hygienekontrollen …, die Pflasterung und Säuberung öffentlicher Verkehrswege, die Trockenlegung stehender Gewässer aber auch die Haltung von Nutztieren und die Platzierung von Misthaufen bildeten wichtige Regulierungsgegenstände“ [2, 3].

Während dieser Blick sich stark auf die ökologischen Merkmale der materiellen Umwelt richtet, kommen mit den Sozialmedizinern des 19. Jahrhunderts auch die sozialen Merkmale des städtischen Lebens in den Blick. Gemeinsam ist beiden Blickfelderweiterungen, dass die Verantwortung des Staates akzentuiert wird.

Neumann [4] beispielsweise erklärt in seiner Schrift „Zur öffentlichen Gesundheitspflege“: „Die Wissenschaft des socialen Lebens ist … die Grundlage der öffentlichen Gesundheitspflege“. Seine auf diesen Grundsatz gerichteten Arbeiten dienten dem sozialen und hygienischen Umbau der Stadt Berlin, deren Stadtverordnetenversammlung er 1858–1905 angehörte. In einer Reformschrift forderte er „ein Recht für alle, eine Gesundheit für alle, eine gleiche für jeden Menschen“ [5].

Eine umfassende Würdigung historischer Blicke auf Hamburg und Altona [6] beginnt mit der Darstellung eines Berichts von 1801 des Arztes Johann Jakob Rambach. Hierzu heißt es: „Der, in eigener Sicht, erste medizinische Topograph Hamburgs verbindet hiesige Gesundheit und Krankheit mit der Schilderung der physischen und sozialen Lebensumstände in der Stadt und bereitet auf diese Weise weitergehenden Ansätzen den Weg“ [6]. Die späteren Berichte über die Gesundheitssituation Hamburgs wurden auch stark beeinflusst von den seit 1831 in Hamburg immer wieder auftretenden Choleraepidemien. Der in dieser Serie letzte umfassende Blick auf die Gesundheitsverhältnisse Hamburgs von 1928 trägt den Titel „Hygiene und Soziale Hygiene in Hamburg“ [7].

In der Nachkriegszeit traten die Erfolge der naturwissenschaftlichen Medizin in den Vordergrund und die Handlungsorientierung von Politik und Verwaltung verengte sich bis ca. Ende der 1970er-Jahre darauf, diese der Bevölkerung zuteilwerden zu lassen.

In den vergangenen 40 Jahren vollzog sich jedoch eine Wende, die den ökologischen und sozialen Lebensbedingungen (insbesondere in Städten) wieder den Stellenwert beimisst, der ihnen für Gesundheit zukommt. Diese Blickfelderweiterung wäre nicht möglich gewesen ohne grundlegende konzeptionelle Neuorientierungen.

Ziel des Beitrags ist es, die Entstehung solcher Neuorientierungen nachzuzeichnen und ihr Verhältnis zueinander zu klären. Ein weiterer Abschnitt geht auf das Konzept „Nachhaltige StadtGesundheit“ (Sustainable Urban Health) ein, das durch eine Hamburger Initiative exemplarisch illustriert wird. Abschließend wird resümiert, in welchen Politik- und Handlungsprogrammen das Konzept StadtGesundheit – aktuell und zukünftig – eine Rolle spielen kann.

## Blickfelderweiterung in den Konzepten der Weltgesundheitsorganisation (WHO) und deren Rezeption

Die konzeptionelle Entwicklung von Programmen der Weltgesundheitsorganisation verläuft über internationale bzw. Weltversammlungen und -konferenzen und deren Entschließungen [8].

### Intersektorale Politik: Alma-Ata-Deklaration zur Primären Gesundheitsversorgung 1978

1978 wurde auf der von WHO und UNICEF veranstalteten Internationalen Konferenz zur Primären Gesundheitsversorgung ein erster Schritt getan, die alleinige Zuständigkeit des medizinischen Sektors für Gesunderhaltung zu überwinden. An zentraler Stelle wird in der Abschlussdeklaration von Alma-Ata [9] hervorgehoben, dass zum Erreichen der Gesundheitsziele das Zusammenwirken von sozialen und ökonomischen Sektoren *außerhalb* des Gesundheitssektors nötig ist. Hintergrund ist die immer deutlicher werdende Ungleichheit des Gesundheitszustands zwischen Industrie- und damals sogenannten Entwicklungsländern, aber auch innerhalb von einzelnen Ländern. Die „Primäre Gesundheitsversorgung“ soll gewährleisten, dass das Ziel „Gesundheit für Alle“ erreicht wird. Das „für Alle“ steht dabei für den Anspruch sozialer Gerechtigkeit und Chancengleichheit für alle Menschen in allen Ländern.

In Punkt VII, 4 der Deklaration von Alma-Ata werden zum ersten Mal explizit andere Politiksektoren angesprochen: Multisektorale Politik „… bezieht neben dem Gesundheitsbereich …, insbesondere Landwirtschaft, Viehzucht, Ernährung, Industrie, Bildung, Wohnungsbau, öffentliche Arbeiten, Kommunikation [ein], und setzt sich für aufeinander abgestimmte Anstrengungen in all diesen Bereichen ein“ [9].

Ein weiteres neues Element ist die Forderung nach Partizipation der Bevölkerung – ein Prinzip, das sich am ehesten lokal umsetzen lässt. Die ein Jahr später stattfindende 32. Weltgesundheitsversammlung (1979 in Genf) proklamierte die Globalstrategie „Gesundheit für Alle 2000“ [10] und machte damit die Bedeutung einer über den Gesundheitssektor hinausreichenden Politikgestaltung für *alle* Länder deutlich. Unter den auf Alma-Ata und die 32. Weltgesundheitsversammlung folgenden konzeptionellen Entwicklungen [11] war – hinsichtlich seiner Resonanz in lokaler Praxis und Politik – die Einrichtung eines WHO-Programms zur Gesundheitsförderung der nächste Meilenstein.

### Gesundheitsfördernde Gesamtpolitik: Ottawa-Charta 1986

Mit der Ottawa-Charta von 1986 entstand ein neues Paradigma, das sich deutlicher als zuvor vom reinen medizinischen Modell verabschiedet hat. Der Begriff „Gesundheitsförderung“ ist dabei nicht einfach eine modernere Bezeichnung für Gesundheitserziehung, sondern hat strategische Bedeutung als Konzept zur Umsetzung der Globalstrategieziele „Gesundheit für alle bis zum Jahr 2000“. Gesundheitsförderung wurde ursprünglich vom WHO-Regionalbüro Europa angeregt und mit Umsetzungskonzepten unterlegt; später wurde das Konzept vom WHO-Hauptbüro als überregionales Projekt übernommen.

Neben anderen neuen Konzepten in der Ottawa-Charta, aber auch übergreifend wird *gesundheitsfördernde Gesamtpolitik* (Build Healthy Public Policy) als eine Schlüsselstrategie für ein umfassendes gesundheitsförderliches Handeln des politisch-administrativen Systems eingeführt und mit folgenden Worten charakterisiert: „Gesundheitsförderung beinhaltet weit mehr als medizinische und soziale Versorgung. Gesundheit muss auf allen Ebenen und in allen Politiksektoren auf die politische Tagesordnung gesetzt werden. … Eine Politik der Gesundheitsförderung muss Hindernisse identifizieren, die einer gesundheitsgerechteren Gestaltung politischer Entscheidungen und Programme entgegenstehen. Sie muss Möglichkeiten einer Überwindung dieser Hemmnisse und Interessengegensätze bereitstellen“ [11].

Für das Prinzip Build Healthy Public Policy schließt öffentliches Handeln (Public Policy) nicht nur den Staat, sondern auch marktwirtschaftliche und zivilgesellschaftliche Akteure ein.

Von unmittelbarer Bedeutung für Städte ist das etwa gleichzeitig mit der Ottawa-Konferenz gestartete internationale Projekt „European Healthy Cities Network“ [12], welches auf die lokale Ebene setzt [13] – die alltägliche Umwelt –, „dort wo … [die Menschen] spielen, lernen, arbeiten und lieben“ [11]. Dieses Projekt ist inzwischen in der WHO-Struktur ein Teil von Urban Health, in dem weitere Teilthemen von Stadt und Gesundheit bewegt werden – kürzlich zum Beispiel mit einer Publikation „A multilevel governance approach to preventing and managing noncommunicable diseases: the role of cities and urban settings“ [14].

### Gesundheit in allen Politikbereichen: Helsinki-Konferenz 2013

Der wichtigste in der Ottawa-Charta genannte Aktionsbereich für Gesundheitsförderung war Build Healthy Public Policy. Er wurde gefolgt von einem gleichsinnigen Konzept, das weniger metaphorisch fordert, Gesundheit müsse in allen Politikbereichen ein Gestaltungskriterium sein: Health in All Policies (HiAP). Im Kontext der WHO wurde dieses Konzept auf der 8. globalen Konferenz in Helsinki, in Zusammenarbeit mit dem finnischen Ministerium für Soziales und Gesundheit endgültig auf die internationale Tagesordnung gesetzt und im Helsinki-Statement festgeschrieben [15].

Bereits 2007 hatte eine EU-Konferenz in Rom „Health in All Policies“ (Untertitel: „Achievements and Challenges“) im Rahmen der finnischen EU-Präsidentschaft auf die politische Ebene gehoben [16]. Und neben den internationalen Nachfolgekonferenzen nach Ottawa [17] ist als direkter Input für Helsinki auch die Expertenkonferenz von WHO und Government of South Australia (International Meeting on Health in All Policies) in Adelaide 2010 zu erwähnen [18]. Hintergrund hierfür war die Planung, HiAP in Südaustralien umzusetzen [19].

Die Helsinki-Konferenz und das gleichnamige Statement zu Gesundheit in allen Politikbereichen beziehen sich explizit auf die Alma-Ata-Deklaration und die Ottawa-Charta und sehen das Konzept verstärkt durch hochrangige Politikdokumente, insbesondere zu nachhaltiger Entwicklung bzw. den Sustainable Development Goals (SDGs, vormals Millennium Development Goals, MDGs).

Im Dokument wird gefordert, das gesamte Regierungshandeln auf Gesundheit zu verpflichten („to engage ‚the Whole of Government‘ in Health“). Als Hintergrund für die Forderung werden die aktuell besonders bedeutsamen Determinanten der Gesundheit genannt (demografischer Wandel, schnelle Urbanisierung, Klimawandel und Globalisierung), die über die Verantwortung und den Einfluss des Gesundheitssektors hinausgehen.

Das zusammen mit der Helsinki-Erklärung veröffentlichte „Health in All Policies – Framework for Country Action“ verlangt die Zusammenarbeit von Regierungen mit der Zivilgesellschaft („Einbeziehen von Gemeinschaften, sozialen Bewegungen und der Zivilgesellschaft“; [15]). Obwohl das Konzept Health in All Policies zunächst für Nationalregierungen formuliert wurde, gilt es ebenso wie „Healthy Public Policies“ auch für die Stadtebene [20].

### Begriffsvarianten der Strategie „Health in All Policies“

Die genannten Entwicklungen, Dokumente und Konzepte wollen theoretische Fundierung für praktisches Handeln sein. Jedoch stammen sie eher aus einem Politikzusammenhang als einem wissenschaftlichen Kontext: „sie zeigen Überlappungen oder werden inkonsistent verwendet“ [21]. So wird beispielsweise im Adelaide-Statement [18] für „Health in All Policies“ auch der Ausdruck „Mainstreaming Health“ verwendet.

Kickbusch hat 2010 den Oberbegriff „Horizontal Health Governance“ für die weiter vorn beschriebenen Kernkonzepte benutzt und in dem Zusammenhang *intersektorale Aktionen, gesundheitsfördernde Gesamtpolitik* sowie *Gesundheit in allen Politikbereichen* als drei Wellen vorgestellt, die jeweils im Kontext ihrer Zeit zu verstehen sind [22]. Intersektorale Aktionen werden dort mit einem rationalen Politikmodell, gesundheitsfördernde Gesamtpolitik mit einem inkrementalistischen Politikstil des Muddeling Through (zu Deutsch: Durchlavieren) und Gesundheit in allen Politikbereichen mit einem Netzwerkansatz politischen Handelns verknüpft. Jedoch sind diese drei Wellen nicht stringent abgrenzbar; sie markieren Akzente, die alle noch Gültigkeit haben.

Der Oberbegriff einer *horizontalen* Politikintegration lässt die Notwendigkeit *vertikaler* Politikintegration, also von der kommunalen bis zur internationalen Ebene, unberücksichtigt, die in den Konzepten durchaus enthalten ist, insbesondere wenn die Umsetzung auf kommunalpolitischer Ebene, d. h. in Städten und Gemeinden, gefordert wird.

Seitens der WHO wurde versucht, in einem Glossar den WHO-Gebrauch der Begriffe zu klären [23]. Zusammenfassend lässt sich daraus schließen:

- Mehrfach werden relevante Ausdrücke, wohl zur wechselseitigen Verstärkung, nebeneinander gestellt ohne erkennbar unterschiedliche Bedeutung, so z. B. „Multisectoral Approaches“, „Health in All Policies and Whole of Government Approaches“ [23].
- Whole-of-Society Approach umfasst: „… individuals, families and communities, intergovernmental organizations and religious institutions, civil society, academia, the media, voluntary associations and, where appropriate, the private sector and industry …“. Während hier und an einigen weiteren Stellen der Begriff Whole-of-Society die Zivilgesellschaft als komplementären Akteur zu Regierungen (Whole-of-Government) umfasst, gibt es aber auch ein Verständnis als Oberbegriff, der alle (staatlichen und nichtstaatlichen) Akteure meint [24].

Ein Teil der Unübersichtlichkeit ist darauf zurückzuführen, dass der Sektorbegriff (auch im Englischen) in unterschiedlichen Bedeutungen gebräuchlich ist: erstens als Ausdruck für *fachliche Spezialbereiche des politisch-administrativen Systems *und zweitens in der *Dreiteilung der Ökonomie in Markt, Staat und den sog. Dritten Sektor*, der die Zivilgesellschaft umfasst [21]. In der Versorgungsforschung sind, in einer dritten Bedeutung, die *Sektoren des Gesundheitssystems* gemeint, also ambulant, stationär etc.

Insgesamt zeigt der Überblick, dass die Begriffsvielfalt zu großen Teilen auf Begriffskonjunkturen zurückzuführen ist.

Etwas außerhalb der bisher referierten Begrifflichkeiten (aber eng verknüpft mit dem Leitprinzip Multisektoralität) steht „nachhaltige Gesundheit“. Der Begriff taucht Ende der 1990er-Jahre im Nachfolgeprogramm von „Gesundheit für alle“ auf und wird für die WHO-Region Europa adaptiert [25]. Mit dem Titel „Gesundheit21“ wird deutlich sichtbar der Schulterschluss mit der Agenda 21 für nachhaltige Entwicklung gesucht und das Gesundheitsthema mit dem Nachhaltigkeitsthema verknüpft: Die vormals getrennten Bereiche „gesundheitsfördernde Lebensweisen“ und „Umwelt“ sind nun in einem gemeinsamen Kapitel zusammengefügt. Unter der Überschrift „multisektorale Strategien für nachhaltige Gesundheit“ wird als Grundsatzziel formuliert: „durch Förderung einer gesunden Umwelt und durch Erleichterung gesundheitsbewusster Entscheidungen die Möglichkeiten für nachhaltige Gesundheit zu schaffen“ [25].

Der im Text mehrfach auftauchende Begriff „nachhaltige Gesundheit“ wird dort allerdings nicht näher erläutert und in späteren Dokumenten nicht weiter verwendet [26]. Das Konzept „nachhaltige *Stadt*Gesundheit“ (Sustainable Urban Health) greift diese Idee, dass Nachhaltigkeit und Gesundheit in einer integrierten Programmatik verfolgt werden müssen, wieder auf.

## Das Konzept „Nachhaltige StadtGesundheit“

In welchem Maße die Themen „Gesundheit“ und „Krankheit“ mit allen gesellschaftlichen Sektoren verwoben sind, illustriert die aktuelle Coronakrise auf eindrückliche Weise. Auch außerhalb einer solchen Pandemie erfolgen umfangreiche Anstrengungen für Förderung, Schutz und Wiederherstellung der Gesundheit. „StadtGesundheit“ (Urban Health) bezeichnet den Versuch, im Gegenstrom zur Vielzahl von Differenzierungen einen integrierenden Blick auf die vielfältigen Strukturen und Prozesse zu werfen. Als Motive zur Bearbeitung dieses Praxis- und Forschungsfeldes sind u. a. weltweite Urbanisierung und sonstiger globaler Wandel zu nennen; zudem bietet sich die Chance, nach 200-jähriger Geschichte von Public Health als Wissenschaft und Praxis gewissermaßen zu den Wurzeln zurückzukehren und erneut die Bedeutung sozialer, physischer und politischer Lebensverhältnisse für Gesundheit zu erkennen [27]. Die Verflechtung menschlicher Gesundheit mit ökologischer und sozialer Nachhaltigkeit im Sinne der o. g. United Nations-Nachhaltigkeitsziele gibt Anlass, ein Konzept von nachhaltiger StadtGesundheit zu verfolgen.

Das übergeordnete Ziel von „Nachhaltiger StadtGesundheit“ ist es, Gesundheit und Nachhaltigkeit in der Stadtgesellschaft zu fördern, u. a. durch wissenschaftlich fundierte Anregungen an die Akteure von Stadtentwicklung und Stadtpolitik [28]. Die deskriptive Zielsetzung ist darauf gerichtet, die Vielfalt des Gesundheitsgeschehens in der Stadt und seine Verbindungen zum Thema Nachhaltigkeit aufzuzeigen („Blickfelderweiterung“). Analytisch geht es u. a. um Erkenntnisse, welche die Grenzen traditioneller gesundheitspolitischer Felder überschreiten und übergeordnete Zusammenhänge erkennen lassen. Beispielsweise wurden die gesundheitlichen Folgewirkungen innerstädtischer Mobilitätsprozesse jahrzehntelang teilweise ausgeblendet. Das handlungspraktische Ziel liegt darin, bestehende Ansätze guter Praxis zu kreieren oder zu identifizieren, um daran anknüpfend neue, nachhaltige „Brücken“ zwischen wissenschaftlichen Disziplinen, gesellschaftlichen Sektoren, AkteurInnen und Handlungsebenen zu bauen, z. B. durch zeitgemäße (Informations‑)Werkzeuge und Kooperationsstrukturen.

Diese Konzeption geht zurück auf unterschiedliche Entwicklungsstränge, von denen zahlreiche bereits benannt wurden. Aus dem internationalen Raum zusätzlich erwähnt sei die New Yorker Academy of Medicine, die das Thema StadtGesundheit seit ihrer Gründung 1847 verfolgt, die humanökologische Gesundheitstradition 1947 als „Ecology of Health“ behandelte und heute mit einem eigenen Institute of Urban Health ausgestattet ist. Inzwischen ist Urban Health ein Forschungs- und Wissensgebiet mit spezifischen Forschungsprojekten, Fachliteratur, Konferenzserien und Programmen, u. a. bei der WHO (z. B. Knowledge Network on Urban Settings, KNUS) und der Europäischen Union; auch in Deutschland entstanden Konzepte, Projekte, Netzwerke und Veranstaltungen (s. hierzu Übersichten, Kap. 5 in [28]).

An dieser Stelle sei ein Blick auf die erwähnten Leitprinzipien „Blickfelderweiterung“ und „Brückenbau“ geworfen (s. ausführlicher Kap. 6 in [28]). Blickfelderweiterung bezieht sich u. a. darauf, neben den Einflüssen *auf* Gesundheit (sog. Gesundheitsdeterminanten) auch die Folgewirkungen *von* eingeschränkter Gesundheit auf das gesellschaftliche Leben in den Blick zu nehmen. „Praktisch alle Stadtsektoren … sind … von den Folgewirkungen eingeschränkter Gesundheit betroffen“ [28], beispielsweise durch eingeschränkte Lernfähigkeit, Arbeits- und Erwerbsfähigkeit, Verkehrstüchtigkeit, psychische Belastbarkeit sowie Teilhabe an Kultur und Wirtschaftsleben. In welchem Maße diese Auswirkungen das gesellschaftliche Leben einschränken können, erleben wir in der gegenwärtigen Pandemiesituation.

Ein weiterer Aspekt von Blickfelderweiterung betrifft die Auswirkungen der medizinischen und pflegerischen Versorgung auf Ressourcen (z. B. Energiemanagement) und Emissionen (z. B. Arzneimitteleinträge in die Oberflächengewässer). (Nicht‑)Nachhaltigkeit im Gesundheitswesen ist bisher ein Nischenthema; Ansätze zur Veränderung sind selten. Thematische Erweiterung von Berichterstattung sowie Fortschreibung von Qualitätskriterien inner- und außerhalb des Gesundheitssektors könnten Abhilfe schaffen. Die genannte Blickfelderweiterung bezieht sich auch darauf, Lebenswelten (Settings) nicht nur als „Zugangswege“ zu Menschen in bestimmten Lebensphasen und Lebenslagen zu sehen, sondern ausdrücklich auch die Gestaltbarkeit dieser Lebenswelten zum Thema zu machen. Zu erweitern ist auch der Zeithorizont; urbane Planungsprozesse wie Bau- oder Verkehrsplanung treffen langfristige Festlegungen, deren Ergebnisse in Form von Gebäuden oder Verkehrswegen oft für Dekaden positive oder belastende Wirkungen zeigen (zu ausgewählten Bezügen von 18 Stadtsektoren zu Gesundheit und Nachhaltigkeit siehe Tab. 6.1 in [28]).

Brückenbau als zweites Leitprinzip bezieht sich auf die Herstellung neuer sinnvoller Verbindungen zwischen unterschiedlichen Themenbereichen, wissenschaftlichen Disziplinen und gesellschaftlichen Sektoren. Der inzwischen vielzitierte Policy-Zyklus bietet einen Ausgangspunkt. Schon die analytischen Aufgaben einschließlich Monitoring/Surveillance und Berichterstattung bergen – über die erwähnte Blickfelderweiterung hinaus – gute Chancen zum Brückenbau, beispielsweise durch gemeinsame (sektorenübergreifende) oder zumindest gut abgestimmte Berichterstattungen (Gesundheit/Umwelt/Soziales und damit Nachhaltigkeit) und durch weitergehende Analysen, wie z. B. Bedarfsanalysen, Folgeabschätzungen und Evaluationen. Regelmäßig stoßen hier unterschiedliche Akteure aufeinander, deren spezifische Traditionen und „Kulturen“ nach besserem Brückenbau verlangen. Entsprechendes gilt für die Strategieentwicklung auf physischer, psychosozialer und Policy-Ebene sowie für die Umsetzung. Hier wäre Bedarf, in bestehenden Ansätzen wie der auf EU-Recht zurückgehenden Umweltverträglichkeitsprüfung (UVP) und der wenig sichtbaren Gesetzesfolgenabschätzung durch „Brückenbau“ beteiligter Akteure eine deutliche Ausrichtung auch auf Gesundheit herbeizuführen, sie konsequent einzusetzen und Interessenkonflikte konstruktiv zu lösen.

Zur Unterstützung lassen sich unterschiedliche Methoden und Werkzeuge anwenden, darunter Ansätze der Kommunikation und Kooperation, Leitlinien, Checklisten und Referenzwerke (als Übersicht siehe Tab. 6.2 in [28]), Surveillance-Werkzeuge (inkl. Indikatorensätze) und Modellierungsansätze für StadtGesundheit (Strukturmodelle, Modellierungsprojekte und -werkzeuge z. B. für Ortsbewegung per Fahrrad und zu Fuß, Planungsräume).

## Hamburger Initiative für nachhaltige StadtGesundheit

Nachteilig an englischsprachigen Buchpublikationen zu Urban Health ist, dass sie zumeist allgemein gehalten sind; allerdings werden sie oft mit Beispielen angereichert (z. B. [29, 30]). Darstellungen mit umfangreicher lokaler Empirie (z. B. [31] zu Chicago) sind selten zu finden. Für die Entwicklung von StadtGesundheit in Deutschland schien es sinnvoll, parallel zur theoretischen Darstellung auch eine lokale Analyse vorzulegen. Als Studienregion qualifizierte sich der Stadtstaat Hamburg zum einen durch die Ankündigung 2015, eine Strategie „Gesundes Hamburg“ zu entwickeln; zum anderen hatte die Mehrzahl der beteiligten Personen seit mehreren Jahrzehnten in Hamburg die Gestaltung von Gesundheitsförderung und -versorgung teils mitgestaltend, teils forschend begleitet.

Eine initiale Arbeitsgruppe mit VertreterInnen aus Praxis und Wissenschaft erprobte die Blickfelderweiterung am lokalen Beispiel und bereitete sie in einem Übersichtsartikel für die weitere Diskussion auf [32]. Auf Basis dieser Vorarbeit startete der Versuch, nachhaltige StadtGesundheit als Fallstudie Hamburg möglichst umfassend darzustellen. Es entstand eine Buchpublikation, deren aus Einzelbeiträgen zusammengesetzten Kapitel folgende Themenfelder behandeln: historische Entwicklung, Gesundheitssituation, Steuerungsstrukturen, medizinische und pflegerische Versorgung, Rehabilitation und Teilhabe behinderter Menschen, Prävention und Gesundheitsförderung, Stadtpolitik und Gesundheit und als Schlusskapitel integrative Ansätze auf sozialräumlicher Ebene [33].

Themenbreite und Perspektivenvielfalt wurden dadurch erreicht, dass die 100 beteiligten ExpertInnen eine Vielzahl unterschiedlicher institutioneller und Erfahrungshintergründe einbrachten; die meisten von ihnen stammen aus Verbänden, Vereinen und Kammern (16), eine geringere Anzahl aus Behörden und nachgeordneten Einrichtungen (11), dann kleinere Anzahlen aus Einrichtungen der Krankenversorgung und der sozialen Arbeit, aus Universitäten, aus Projekten und Verbünden, schließlich aus Kapitalgesellschaften, insgesamt sind mehr als 70 Institutionen beteiligt.

Zu den vorrangigen Zielen der Buchpublikation gehört es, die Verflechtungen von Gesundheit mit den verschiedensten Sektoren Hamburger Stadtpolitik aufzuzeigen. Eine Übersicht gibt Tab. 1. Für drei Stadtsektoren zeigt Tab. 2 ausgewählte Teilthemen, die in den Beiträgen zur Sprache kommen.

**Table 1:** 

Thema	Buchbeiträge
Arbeitswelt	7.1 Ganzheitliche Gesundheitsförderung von Langzeitarbeitslosen in ihrer Lebenswelt
	7.2 Gesundheitsförderung als Beitrag zur Fachkräftesicherung
	7.3 Arbeitsschutz
Bewegung	7.5 Sportvereine und Gesundheit
	7.8 Mobilität und Verkehr
Bildung	6.2 Bildung, Schule, Jugend
	6.3 Hamburger Volkshochschule und das Thema Gesundheit
	1.7 Hamburger Schülerstudie 1979–1987
Soziales, Integration	3.1 Wohlfahrtsverbände
	4.5 Gesundheitsversorgung abseits des Regelsystems der Krankenversicherung
	4.6 Die medizinische Versorgung Geflüchteter und Asylsuchender
	5.1 Rehabilitation und Teilhabe behinderter Menschen
	5.3 Q8; Prozesse zur Verbesserung der Versorgungstrukturen im Quartier
	6.1 Der Beitrag der Frühen Hilfen, der Familienförderung und der Kindertagesbetreuung zur Gesundheitsförderung
Stadtentwicklung	7.9 Stadtentwicklung und Gesundheit
	7.10 Stadtentwicklungspolitik gestern und heute
Umwelt und Nachhaltigkeit	7.4 Feuerwehr und Katastrophenschutz
	7.6 Lärmbelastungen
	7.7 Luftqualität
	7.11 Stadtklima, Klimawandel und -anpassung
	8.1 Indikatorensystem HEINZ für Hamburgs Zukunftsfähigkeit
Verbraucherschutz	7.12 Gesundheitlicher Verbraucher- und Patientenschutz
	7.13 Gesundheitlicher Verbraucherschutz – Lebensmittelsicherheit
Wirtschaft	7.14 Gesundheitswirtschaft
	7.15 Wohnungswirtschaft und Gesundheit
	3.5 Hafen- und Flughafenärztlicher Dienst
Wissen, Forschung, Ausbildung	7.16 Gesundheitsforschung
	7.17 Vernetzung der Versorgungsforschung
	7.18 Landesforschungsförderung
	7.19 Gesundheitspersonal, Ausbildung und Studium

**Table 2:** 

Thema	Buchbeiträge	Ausgewählte Teilthemen
Arbeitswelt	7.1 Ganzheitliche Gesundheitsförderung von Langzeitarbeitslosen in ihrer Lebenswelt	Verminderung von Vermittlungshemmnissen für den ersten Arbeitsmarkt; Pilotprojekt; subjektive/normative/gesellschaftliche/informatorische Barrieren
	7.2 Gesundheitsförderung: Ein Beitrag zur Fachkräftesicherung	Sicherung von Beschäftigungsfähigkeit; Schaffung guter Arbeitsbedingungen; Projekt „Perspektive Arbeit und Gesundheit“; Arbeitsmarktvorsorgecheck
	7.3 Arbeitsschutz	„Humanisierung der Arbeit“; betriebliche Gesundheitsförderung; Arbeitsschutzgesetz; Hamburger ArbeitsschutzPartnerschaft; Gefährdungsbeurteilung
Stadtentwicklung	7.9 Stadtentwicklung und Gesundheit	Schumachers Federplan; körperliche Gesundheit: Bewegungsräume schaffen; mentale Gesundheit: Freiräume schenken; soziale Gesundheit: Lebensräume bauen
	7.10 Stadtentwicklungspolitik gestern und heute	Landes- und Stadtteilebene; Stadt(teil)entwicklungsprogramme; Gesundheitsförderungsprojekte in den integrierten Entwicklungskonzepten
Wirtschaft	7.14 Gesundheitswirtschaft	Erster und zweiter Gesundheitsmarkt; zwei Gesundheitscluster: Life-Science-Cluster und Gesundheitswirtschaftscluster; Projekt „Aktive und gesunde Quartiere“
	7.15 Wohnungswirtschaft und Gesundheit	Verband norddeutscher Wohnungsunternehmen; Sozialmanagement; Wohnmodelle, z. B. autofrei; Checklisten und Praxisempfehlungen
	3.5 Hafen- und Flughafenärztlicher Dienst	Hamburg Port Health Center; Seegesundheitserklärung; „Schiff geht an die Kette“; div. Bündnispartner auch für Sozialarbeit, maritime Medizin, Hygiene

Zur Abrundung der vorausgehenden, systematisch angelegten Buchabschnitte werden in einem eigenen Kapitel „Integrative Ansätze auf sozialräumlicher Ebene“ beleuchtet, die teils auf die Gesamtstadt, teils auf Bezirks‑, Stadtteil- oder Quartiersebene ausgerichtet sind und neue Wege für das Zusammenspiel der Akteure illustrieren. Hier geht es um ein Indikatorensystem für urbane Zukunftsfähigkeit, ein Netzwerk zu „psychischer Gesundheit“, integrierte Stadtteilentwicklung und Sozialraummanagement, Gesundheits- und Pflegekonferenzen als integratives Instrument, integrative Prävention und Versorgung bei älteren Menschen, ein Modell integrierter quartiersbezogener Gesundheitsförderung („Lenzgesund“), ein Stadtteilgesundheitszentrum und um einen „Gesundheitskiosk“ als Kooperationsschnittstelle medizinischer und sozialer Versorgung.

Im Schlusskapitel diskutieren die Herausgeber die Grenzen des Erkenntnis- und Handlungsansatzes StadtGesundheit sowie weitere Schritte zur „Blickfelderweiterung“ und zum „Brückenbau“ – auch in Verbindung mit anderen zukunftsorientierten Diskussionen. Auf der Suche nach einem geeigneten Rahmen dafür trat die „Patriotische Gesellschaft von 1765 – Hamburgische Gesellschaft zur Beförderung der Künste und nützlichen Gewerbe“ ins Blickfeld [35]. Sie gilt als älteste zivilgesellschaftliche Organisation im deutschen Sprachraum und engagierte sich bereits in früheren Zeiten für gesundheitsrelevante Themen, z. B. einen Rettungsdienst. Aktuell bilden Projekte zur Stadtentwicklung einen Schwerpunkt. Die Patriotische Gesellschaft begrüßte den Vorschlag, das Thema „Nachhaltige StadtGesundheit Hamburg“ mit seiner integrierenden, stadtsektorenübergreifenden Ausrichtung und seinem Fokus auf Mitwirkung an Stadtgestaltung aufzugreifen und richtete hierzu eine Themengruppe ein, die seit Anfang 2019 regelmäßig zusammenkommt [36]. Diese ehrenamtlich arbeitende Gruppe formulierte ein Eckpunktepapier, welches die enge Verbindung von Gesundheit, ökologischer Nachhaltigkeit und sozialer Gerechtigkeit zum Ausdruck bringt, Veränderungsbedarf identifiziert und Ansatzpunkte für die Praxis benennt, z. B. in Form einer Mitwirkung an Gestaltungsprozessen in der Stadtentwicklung.

Um in aktuellen Projekten innerstädtischer Verkehrspolitik die Themen „Gesundheit und Nachhaltigkeit“ zu stärken, entwickelte die Themengruppe eine siebenteilige Posterserie unter dem Motto „Verkehrspolitik ist auch Gesundheitspolitik“. Einzelne Poster behandeln u. a. die sich in Hamburg bietenden Chancen für Gesundheit und Nachhaltigkeit, bestehende Belastungen, Daten zur Gesundheit im Stadtteil sowie programmatische und rechtliche Ansatzpunkte für das Themenfeld „Nachhaltiger StadtGesundheit“. Die Serie wurde in einem Workshop zur StadtGesundheit im November 2019 eingesetzt und ist über die Webseite der Patriotischen Gesellschaft Hamburg abrufbar.

Die Arbeit im ersten Kalenderjahr (2019) wurde in einem Bericht dokumentiert [37]. Danach stießen bisher insbesondere folgende Teilthemen auf große Resonanz in der Themengruppe: (i) Stadtgrün und Stadtblau in ihrer Mehrfachfunktion für Gesundheit, Umwelt, Naturschutz und Nachhaltigkeit, samt Gärtnern in der Stadt in sozialökologischer Perspektive, (ii) Verkehr, Gesundheit und Nachhaltigkeit einschließlich Verkehrsberuhigung, Langsamverkehr und Kombinierbarkeit verschiedener Bewegungsformen, (iii) Metropolregion: Verbindungen zwischen Stadt und Umfeld durch Personen‑, Verkehrs‑, Güter und vielfältige weitere Stoff- und Energieströme. Alle diese Themenfelder sind Gegenstand urbaner Planungs- und Entwicklungsprozesse. Das Spektrum entsprechender Projekte mit Bezug zu Gesundheit und/oder Nachhaltigkeit ist umfangreich und bietet vielfache Ansatzpunkte. Für die weitere Arbeit stehen unterschiedliche Arbeitsformen zur Verfügung, darunter Kommunikation und Bewusstseinsbildung, Aufbereitung von Informationen zu relevanten Teilthemen und Mitwirkung an konkreten Aktionen, z. B. zur Stadtentwicklung. Als nächster Meilenstein ist für 2021 eine zweitägige „Tandemveranstaltung“ geplant (5. Hamburger Symposium zur regionalen Gesundheitsversorgung in Zusammenarbeit mit der 7. Konferenz Stadt der Zukunft), bei der sich zahlreiche Akteure austauschen und weitere Arbeitsschritte entwickeln werden.

## Resümee

Die Idee der engen Wechselwirkungen zwischen menschlicher Gesundheit und Stadt beginnt schon mit Hippokrates’ berühmter „Schrift über die Umwelt“: „Der Arzt, der in eine Stadt kommt, muss nicht nur die Jahreszeit, die Winde, das Wasser, den Erdboden und die geographische Lage des Ortes berücksichtigen, sondern auch die Lebensweise …“ [38]. Einige exemplarische Belege in der Einleitung unseres Beitrages haben gezeigt, wie diese Idee dann vom 17. bis zum beginnenden 19. Jahrhundert gesundheitspolitisch relevant wurde. Die WHO hat mit der Alma-Ata-Konferenz von 1978 eine Renaissance des Denkens über Determinanten der Gesundheit in Politiksektoren jenseits der Medizin eingeleitet und daran anknüpfend in ihren Programmen immer wieder entsprechende Handlungskonzepte in den Vordergrund gestellt.

Bei den beschriebenen WHO-Konzepten (multisektorale/intersektorale Aktionen, gesundheitsfördernde Gesamtpolitik und Gesundheit in allen Politikbereichen) handelt es sich um verschiedene Akzente im Kontext unterschiedlicher Politikkonzeptionen. Von größter Bedeutung scheint uns das Konzept Health in All Policies (HiAP) zu sein, das griffig und politikfähig ist: HiAP hat den Eingang sowohl in die WHO- als auch in die EU-Politik geschafft. Auch ist mit diesem Konzept eine Integration gesundheitsfördernder Politikansätze in den globalen Kontext der Nachhaltigkeitsentwicklung (Sustainable Development Goals) gelungen: „As a concept, HiAP is in line with the Universal Declaration of Human Rights, the United Nations Millennium Declaration, and accepted principles of good governance …“ [15].

In Deutschland hat das Zukunftsforum Public Health HiAP [39] in den Mittelpunkt einer neuen Public-Health-Strategie gerückt. In einem Grundsatzpapier wird ein Verständnis des Konzepts als Doppelstrategie formuliert: „Im Mittelpunkt von HiAP steht die Anforderung einer politikfeldübergreifenden Strategie für Gesundheit. Eine solche Strategie muss eine Doppelstrategie von regierungspolitischen und gesellschaftlichen Ansätzen sein … Whole-of-Government-Ansatz bezieht sich vor allem auf die politische Verantwortung der Regierungen. Doch diese können nur wirksam sein, wenn es auch zivilgesellschaftliches Engagement (Whole-of-Society-Ansatz) gibt. Erst durch das Zusammenwirken beider Ansätze wird ermöglicht, dass die Prinzipien von Health in All Policies … in den Lebenswelten der Menschen wirken und somit der konkrete Alltag über gesundheitsfördernde Rahmenbedingungen … auf Gesundheit und Wohlbefinden ausgerichtet wird“ [40].

Für die Bearbeitung der Wechselwirkungen von Stadt und Gesundheit böte eine verbindliche Public-Health-Strategie den Rahmen für koordiniertes Handeln, das sich bisher überwiegend in wenig verbundenen und zumeist schwach ausgestatteten Initiativen und Programmen abspielt [41]. Als solche sind vor allem zu nennen: das Gesunde-Städte-Netzwerk, das Aktionsprogramm Umwelt und Gesundheit (APUG) auf Bundesebene, das Städtebauförderprogramm Soziale Stadt (ab 2020: Sozialer Zusammenhalt – Zusammenleben im Quartier gemeinsam gestalten) sowie der sogenannte Partnerprozess zur Etablierung Integrierter Kommunaler Strategien der Gesundheitsförderung [42].

All diese Programme und Initiativen verfolgen wichtige Ziele und sind dabei teilweise erfolgreich, aber sie stoßen zu früh an ihre Grenzen. Bedauerlicherweise wird aus der Palette verfügbarer Gesundheitsanalysen erst ein kleiner Teil systematisch genutzt. Dieses Repertoire an spezifischen Methoden und Werkzeugen für nachhaltige StadtGesundheit sollte konsequenter eingesetzt und für künftige Anforderungen fortentwickelt werden, um die Gesundheit städtischer Populationen und urbane Nachhaltigkeit für die Zukunft zu gewährleisten. Um dem vorhandenen Potenzial künftig größere Geltung zu verschaffen, wäre es hilfreich, dem Einsatz solcher Werkzeuge in Aus- und Fortbildung in Gesundheits‑, Planungs- und weiteren beteiligten Disziplinen mehr Raum zu geben. In der Praxis können wir aus guten Beispielen lernen und weiter experimentieren, um geeignete Wege zu beschreiten.
